# Novel microcapsules for internal curing of high-performance cementitious system

**DOI:** 10.1038/s41598-020-65285-6

**Published:** 2020-05-20

**Authors:** Xiaoyu Shang, Baojian Zhan, Jiangshan Li, Rui Zhong

**Affiliations:** 1 School of Civil Engineering and Architecture, Northeast Electric Power University, Jilin, 132012 PR China; 2 Department of Civil and Environmental Engineering, Hong Kong Polytechnic University, Hong Kong, PR China; 3 State Key Laboratory of Geomechanics and Geotechnical Engineering, Institute of Rock and Soil Mechanics, Chinese Academy of Sciences, Wuhan, 430071 PR China; 4 Department of Civil and Environmental Engineering, National University of Singapore, Singapore, 117576 Singapore

**Keywords:** Engineering, Materials science

## Abstract

Conventional internal curing materials for high-performance cementitious system cannot easily have artificial modifications, such that the curing effect is difficult to control during the process. In this study, a novel microcapsule is proposed for controlled internal curing of cement-based materials. The microcapsules are synthesized by a double emulsion method to form a polymer shell-water core structure. The sensitivity of polymer shell to alkaline environments is used to trigger the release of core water. Thus, water release can be controlled by tailoring the shell thickness and microcapsules sizes by changing the polymer dosage and stirring rate during synthesis. The experimental results indicate that the novel microcapsules can effectively release water for internal curing of a cementitious matrix, which exhibits a high curing efficiency in terms of nearly autogenous shrinkage and increases the compressive strength. The novel microcapsules could be promising internal curing agents to enhance high-performance cement-based materials.

## Introduction

Increasingly high performances of concrete materials are needed to satisfy the ever-increasing requirements of infrastructure construction. Currently, the compressive, tensile and flexural strengths of high-performance concrete range from 200 to 800 MPa, 25 to 150 MPa and 30 to 141 MPa, respectively^1^. However, self-desiccation and high sensitivity to early-age cracking is an important phenomenon in a high-performance cementitious system. Internal curing (IC) has been widely employed to mitigate cracking and shrinkage^2–4^. Although considerable success has been achieved using super absorbent polymers (SAPs)^3,4^ and pre-wetted lightweight aggregates (LWAs)^5,6^, these materials have some deficiencies that need to be addressed.

- Voids or deflects left IC agents, especially for SAPs, decrease the strength of concrete^5^.
- The mixture design must be precisely controlled to compensate absorption/desorption with dry SAPs^5^.
- Excessive rapid water release during initial setting significantly affects the strength and shrinkage of concrete, especially when LWAs are used^7^.
- morphology of LAWs and SAP create dispersion problem in cement-based materials^8^.

In general, it is difficult to modify conventional IC materials, such as LAWs and SAPs, in terms of their absorption/desorption ability and dispersion in concrete to control curing action^7^. In practical situations, internal water curing is facilitated by small inclusions dispersed in concrete that contain water: this water is retained during mixing and up to the setting time and released during cement hydration^9^. Thus, intelligent materials could be used to produce more effective internal curing condition.

Encapsulation is a significant technique in many fields of biology engineering^10,11^, medicine engineering^12^, cosmetics production^13^, food engineering^14^. Microcapsule synthesis has been shown to be feasible for encapsulating an aqueous phase^15,16^. External damage to the microcapsules releases core water into a cementitious matrix for internal curing. However, the release of curing water in advanced of the final setting of the cementitious matrix negatively affects the workability and strength of concrete. If the curing water is released too slowly, autogenous shrinkage results^4,7^. Moreover, the release of water from microcapsules leaves voids in the cement matrix that deteriorate its mechanical properties^3^. In view of this, an alkaline environment is an effective trigger for the release of internal curing water. The sensitivity of synthesized microcapsules to alkaline environment can be exploited to spontaneously release curing water. Thus,

it is important to synthesis a kind of intelligent microcapsules that release curing water at an appropriate time. This procedure can overcome the drawbacks of current IC agents. Microcapsules have been reported to be sensitive to chemical, biological and physical triggers. However, methods for controllably release water are still in investigation^17^. Such methods have promising potential application to cementitious materials.

**Figure 1:**
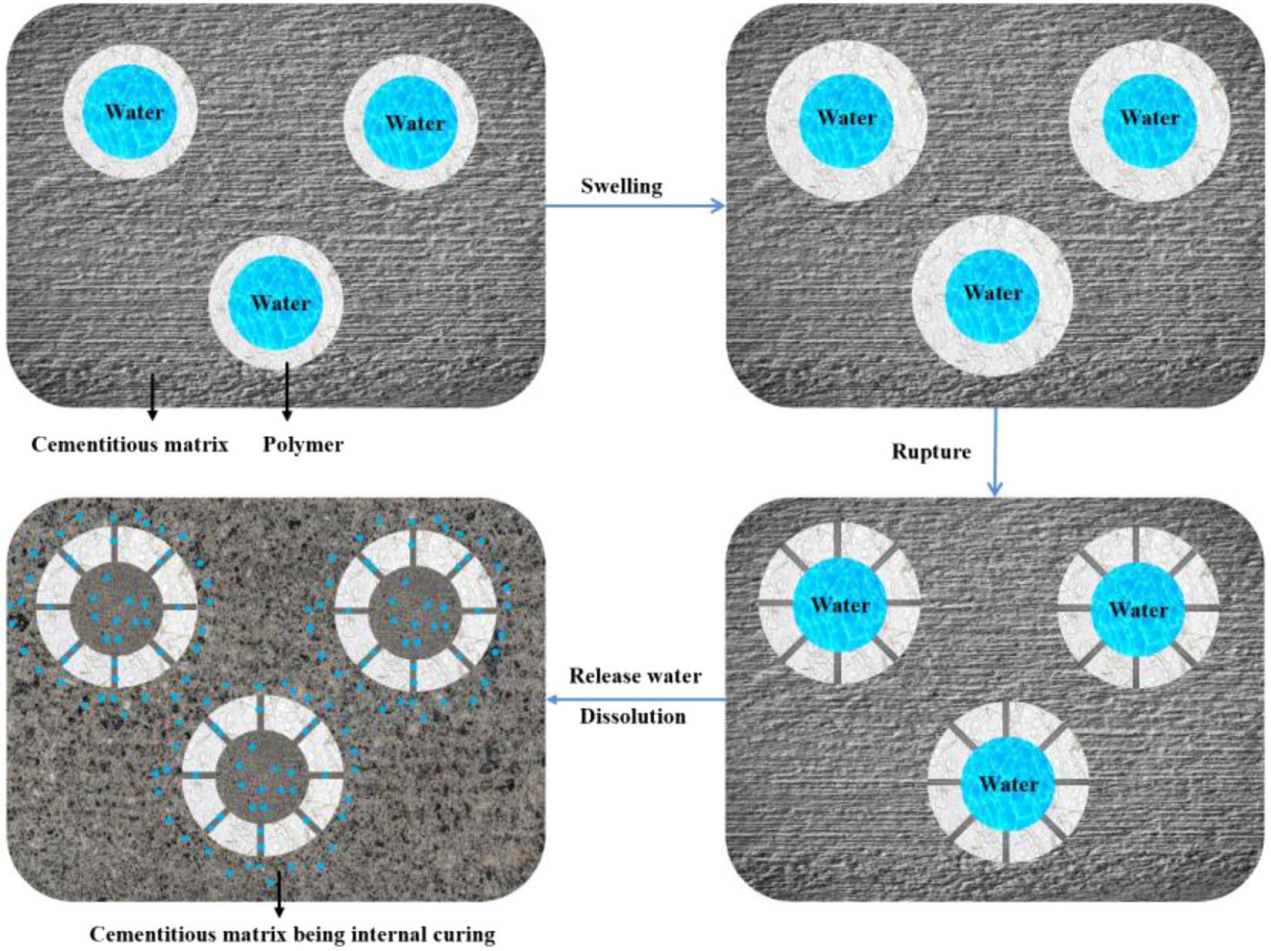
Mechanism of water release from microcapsules into cementitious matrix.

In this study, a novel microcapsule was synthesized to realize controlled internal curing for cement-based materials. A novel emulsions method was used to prepare microcapsules with a water core-polymer shell structure. The external shell was made of polymethyl methacrylate (PMMA), an alkali-sensitivity material. Swelling and rupturing of the shell in saline condition (cement-based materials) triggered the release of water into the cementitious matrix. The time of water release was artificially controlled by tailoring the shell thickness and dimension of the microcapsules by varying the emulsion process parameters. Figure 1 presents a schematic of release mechanism of the microcapsules-matrix system. The microcapsules experienced swelling, rupture, release water and dissolving as the cementitious matrix hardening. Controlled release of the core water to the surroundings occurred over 5-26 h, where more than 90% of the core water was continuously release in 12 h.

## Result

### Characteristics of microcapsules

**Figure 2:**
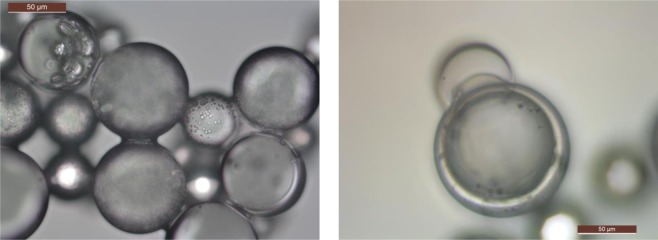
OM image of (**a**) microcapsules in water and (**b**) crushed microcapsules after nitrogen freezing. Capsule fabrication: polymer dose, 1%; stirring rate, 250 rpm; PVA concentration in outer water phase, 0.5%.

**Figure 3:**
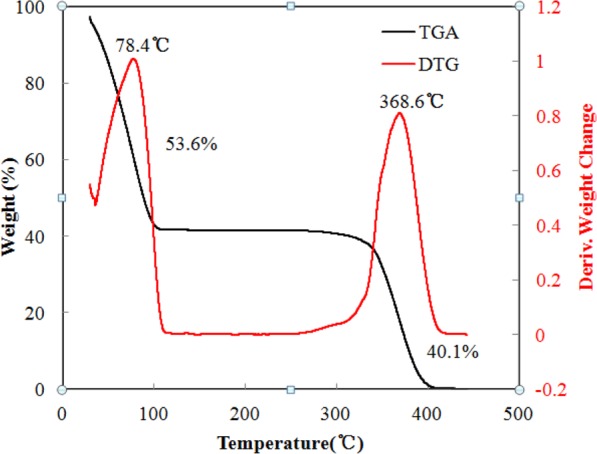
TGA and DTG curves for microcapsules.

**Figure 4:**
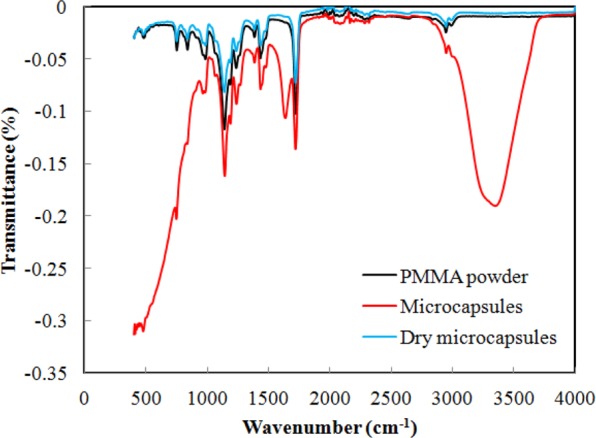
FTIR curves for micocapsules.

Figure 2(a) shows the OM image of the microcapsules in the water, revealing almost spherical particles with smooth surfaces. Figure 2(b) is an image of the cross-section of microcapsules, where a cavity can clearly be observed in the centre of the microcapsule, confirming the core-shell structure. The average particle size and shell thickness are approximately 116.1 μm and 4.4 μm, respectively. The TGA and DTG curves (Fig. 3) were used to determine a weight loss of 55.87% at 100 °C from the evaporation of physically filled water inside the microcapsules, that is, the microcapsule core was 55.87% water. The weight losses of 41.36% from 100 °C to 400 °C was mainly due to a sudden drop in the weight from 342.62 °C to 391.17 °C form the thermal decomposition of the PMMA shell. The result show that the polymer shell showed is more thermostable than the aqueous core. Figure 4 shows the FTIR curves for the microcapsules, the dry microcapsules and the PMMA powder. There is no difference between the dry microcapsules and PMMA powder, which indicates that the process of double emulsion made no changes to the functional group of the raw materials. The peak in the microcapsule spectrum in the 3000–3500 cm^−1^ range is associated with the OH from the water emulsified into microcapsules.

### Water release behavior

**Figure 5:**
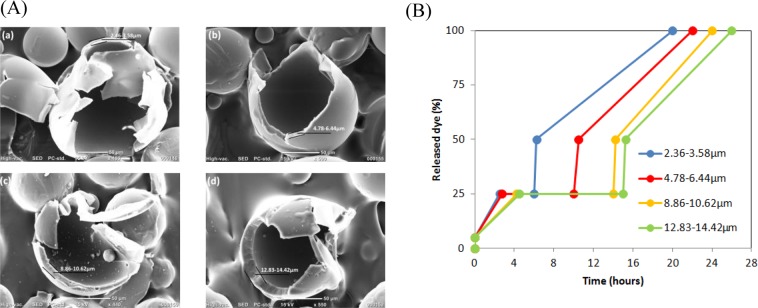
(**A**) SEM images of microcapsules fabricated using different polymer doses: (a) 1%; (b) 3%; (c) 5%; and (d) 7%; preparation conditions: stirring rate: 200 rpm, PVA concentration in outer water phase: 0.5%. (**B**) water release time for different microcapsules shell thickness.

**Figure 6:**
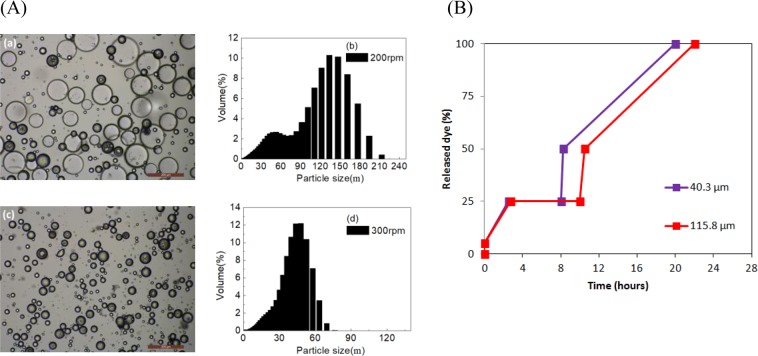
(**A**) OM images and particle size distribution of microcapsules fabricated under different stirring rate: (a,b) 200 rpm; (c,d) 300 rpm; preparation conditions: polymer dose: 5%; PVA concentration in outer water phase: 0.5%. (**B**) water release time for different microcapsules shell thickness.

The water release behaviour was quantitatively monitored by UV-Vis absorbance, as shown in Figs. 5(B) and 6(B). Around 5% of methylene-blue (which was used as an indicator for the encapsulated water) was quickly released after microcapsules were dispersed in sodium hydroxide solution (pH 13). Such burst release was induced by the difference in the ionic strengths inside and outside the microcapsules^18^. The high sodium hydroxide concentration in the external solution greatly facilitated water permeation through the tortuous network in the shell. Once the gradient was quickly disappeared, the dye concentration in the media remained relatively stable for 6 hours. In fact, the swelling degree of alkaline-sensitive PMMA increase slowly during this period. After 6 hours, this continuous swelling resulted in tiny breaks on PMMA shell, and the gradual release of the dye (with the encapsulated water) form the microcapsules was observed. dye (or encapsulated water) was observed to be gradually released from microcapsules. The microcapsules released over 90% of the core water in 24 h.

The results showed that the water release behaviour depended on the shell thickness and microcapsule size, which essentially means that water release behaviour can be controlled by adjusting the shell thickness and size. The typical fabrication parameters of the polymer dosage and the stirring rate were varied to produce microcapsules with different sizes and shell thicknesses. Figures 5 and 6 show microcapsules fabricated with different polymer dosage and stirring rate. The Polymer dose is a key factor influencing the shell thickness. Figure 5(A) shows SEM images of microcapsules fabricated using different polymer dosages. The shell thickness increases from 2.36 µm to 14.42 µm as the polymer dosages increases from 1% to 7%. Figure 5(B) shows that the initial water release was clearly delayed as the shell thickness increased. Initially, 5% dye was rapidly released. The quantity of released dye then remained stable at 25%, where the duration of stable release depended on the shell thickness. The duration of the initial rapid release of dye after microcapsules reached the stable level also increased with the shell thickness. The stirring rate is a determining factor for the microcapsule size. Figure 6(A) shows that the average microcapsule size increased from 40.3μm to 115.8μm as the stirring rate was increased from 200 to 300 rpm. Figure 6(B) shows that large microcapsules released internal curing water more slowly than small microcapsules, which could be attributed to the change in the specific surface areas (SSA) with the particle size. Small microcapsules with high SSA provide more transport pathway for the release of internal curing water. The water content of microcapsules was increased by accelerating stirring. The maximum water content of 74.1% was obtained at a 300 rpm stirring rate. More water was emulsified into microcapsules to promote internal curing. These results indicate that different shell thicknesses and microcapsules sizes were produced by varying the polymer dosage and stirring rate during emulsification. More importantly, the results indicate that water released time can be artificially controlled by varying the shell thickness.

### Characteristics of microcapsule-mortar system

**Figure 7:**
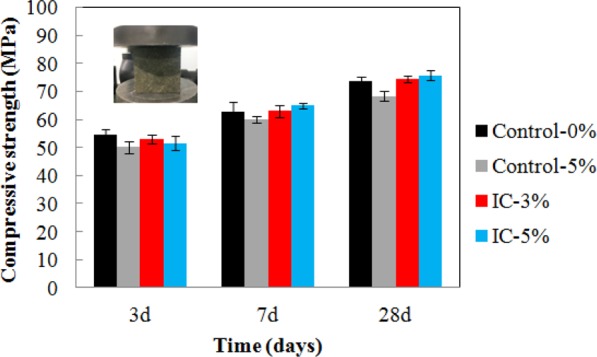
Compressive strength of mortar-microcapsules system at 3, 7 and 28 days.

Figure 7 shows the compressive strength of mortars measured at the curing ages of 3, 7 and 28 days. The mortar compressive strength increased with the hydration age, regardless of the presence of the water released by the microcapsules. The mortar compressive strength generally decreased as the quantity of microcapsules increased at the hydration age of 3 days. This result was attributed to the cavity structure and low strength of microcapsules. However, after curing for 7 and 28 days, the compressive strength of mortar containing microcapsules became comparable to that of the control mortar, especially for the one with 3% internal curing water. The compressive strength of mortar with internal curing water was enhanced by 10% over that of the control mortar without internal curing water because the water released by microcapsules promoted cement hydration.

The detrimental effect of IC materials with poor mechanical properties on the compressive strength of cement paste has been previously reported^19^. However, these results indicate that this effect could be eliminated by optimizing the structure and content of internal curing agents. Jensen and Hansen reported the water entrainment has two opposing effects of water entrainment on strength: spherical macropores in water-entrained cement paste deteriorate strength, while improving curing condition^20^. The strength enhancement may result from more IC efficiency. The strength enhancement could also be attributed to the dispersion and dissolution of micropores filled with calcium hydroxide^5^.

**Figure 8:**
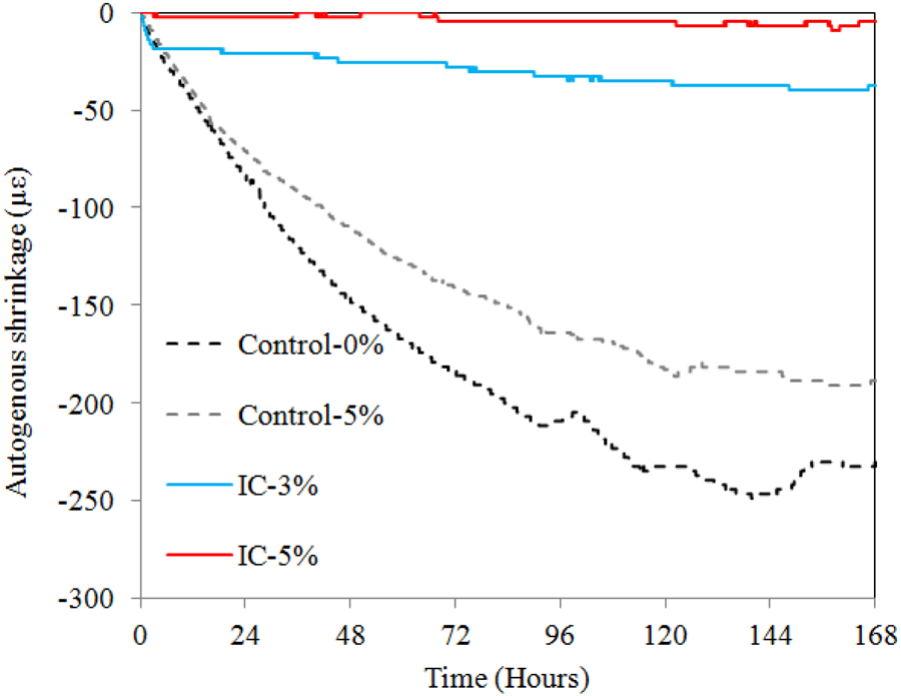
Autogenous shrinkage for mortar-microcapsules system.

The autogenous shrinkage curves are shown in Fig. 8. The autogenous shrinkage of the internally cured samples was significantly improved over that of the control samples. The autogenous shrinkage almost stopped after approximately 24 h. The total autogenous shrinkage was less 40 µε for both internally cured samples. In particular, the autogenous shrinkage of the IC-5% sample was nearly zero. The self-desiccation caused by cement hydration was fully compensated by the internal curing water released by the microcapsules. Thus, cement composites with near zero autogenous shrinkage can be fabricated via internal cuing by microcapsules.

**Figure 9:**
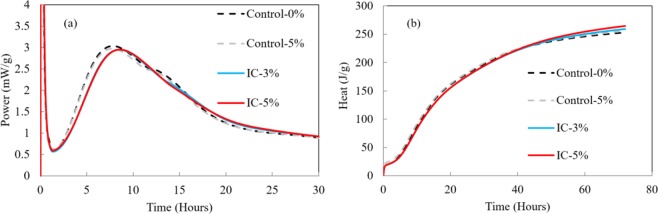
(**a**) Heat evolution rate, and (**b**) total hydration heat; matrix with water/binder = 0.25.

Figure 9. displays the heat evolution of the mortar-microcapsule system. The main peak height is slight reduced upon addition of microcapsules, regardless of whether the core water existing, compared to mortar without microcapsules. It means that microcapsules addition reduces the maximum heat release rate from cement hydration. The rightward peak shift for IC-3% and IC-5% indicates a slightly delay in the cement hydration from the inclusion of microcapsules. Figure 9(b) demonstrates the total hydration heat of the mortar-microcapsules system. At the initial time, the hydration heat process is similar for all the mortar-microcapsules systems, although the control samples exhibited slightly higher hydration heats than the IC samples. Note that at 42 h, the mortar with microcapsules (IC-3% and IC-5%) begin to release higher hydration heat than the control samples (Control-0% and Control-5%). This result may indicate that internal water form the microcapsules is beginning to participate in the hydration reactions. The internally cured concrete releases a higher amount of hydration heat than the control samples, as has been previously reported^7,21^.

**Figure 10:**
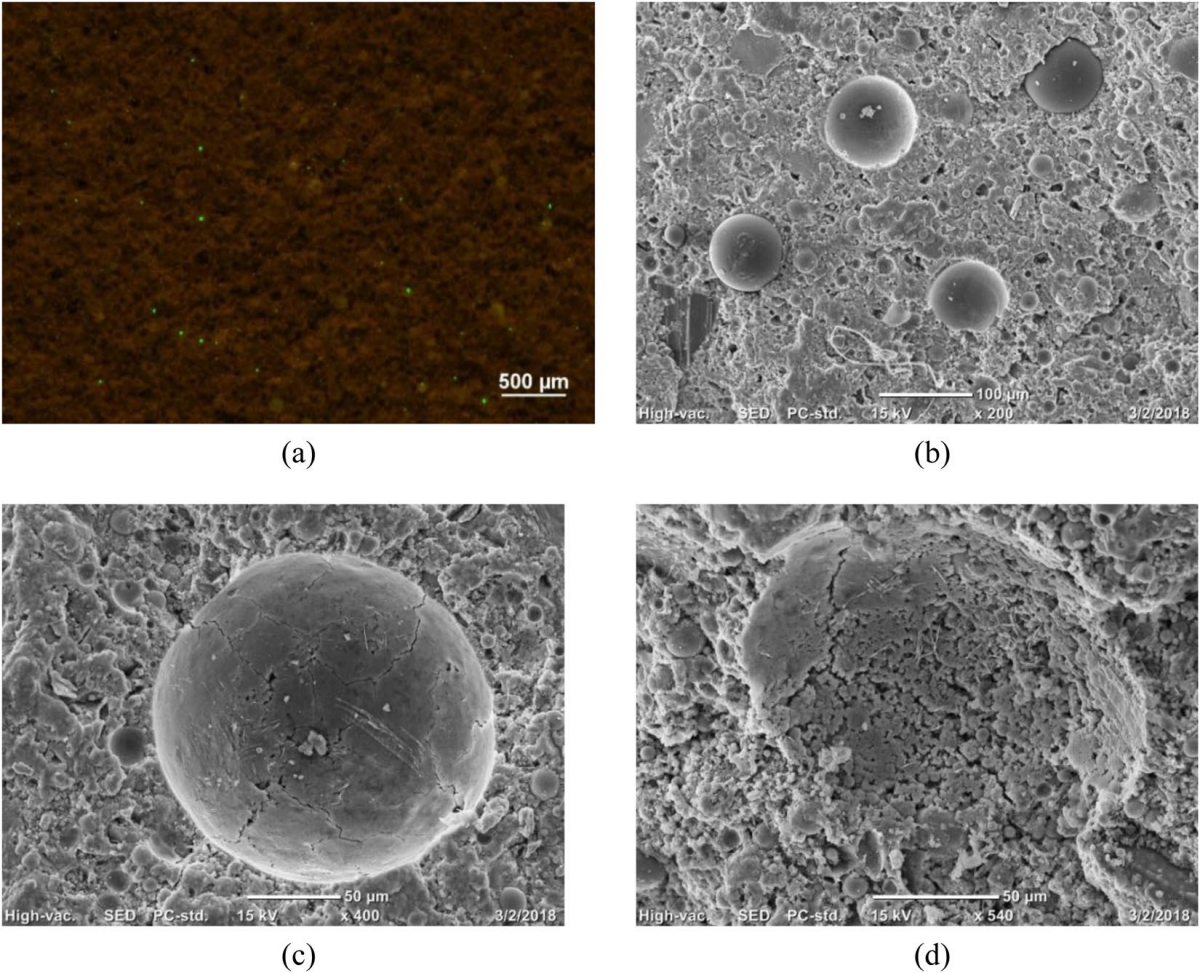
(**a**) Fluorescence image of microcapsules in hardening cementitious matrix for a slice from the IC-5% sample with a field of view is 620 mm × 890 mm; (**b**) SEM image of microcapsules in hardening cementitious matrix; (**c**) SEM image of cracking on microcapsules triggered by cementitious matrix. (d) SEM image of dissolved microcapsules in cementitious matrix; microcapsule fabrication: polymer dose, 1%; stirring rate, 250 rpm; PVA concentration in outer water phase, 0.5%.

Fluorescence microscope was carried out to identify the status of the microcapsules in the cementitious matrix. Figure 10(a) shows that the fluorescent green microcapsules were uniformly distributed in the hardening cementitious matrix as a discrete phase of individual particles. The same results were observed in SEM image, Fig. 10(b). Thus, microcapsules can be well dispersed in cementitious materials after mixing or vibration. The even distribution of microcapsules in a matrix enhances curing of neighbouring matrix with an optimal amount of IC water. Fig. 6Ac shows that microcapsules were also evenly dispersed in the water solution without any additional dispersion method. This result could be attributed to the beneficial morphology, physical and mechanical effects of particles with standardized spherical shapes and smooth surfaces. The angularity of SAP particles results The angularity of SAPs particles results in an inhomogeneous dispersion, especially for small particle size^5^. This observation may explain why SAP application has not been reported for mass concrete.

A schematic for the mechanism of water release from microcapsules into a cementitious matrix is shown in Fig. 1 above. The water release behaviour depends on the swelling and fracture of the polymer shell. The swelling degree of a polymer in an alkaline environment is expected to increase with time. A sufficiently large morphological change of the polymer shell induces shell rupture, thereby completely releasing the water inside the microcapsules within a well-defined time. The mechanism of water release was verified by the SEM images. The SEM images in Fig. 10(c) show that the microcapsules were exposed to the cementitious matrix. The appearance and propagation of cracks were clearly observed on the surface of the microcapsules, reflecting the process of release of the internal water triggered by the saline environment. Figure 10(d) shows the dissolution process after the microcapsules fractured and released the internal water.

## Discussion

Several parameters affect the efficiency of internal curing, such as the quantity, type and size of particles and the water saturation state of the IC agents. However, the recent research indicates that the overriding parameter seems to be the internal structure of the IC agents^22^. Thus, in this study, a novel core-shell microcapsules structure was designed for an internal curing system. These novel microcapsules offer three advantages over conventionally used IC agents.

Conventional IC materials, especially LWAs with large pores, provide excessive free water to the cementitious matrix, which reduces the matrix strength^2^. The extremely high strength of high-performance concrete results from the low water to cement ratio, very low porosities, and extremely small defects in the microstructure. Thus, it is of significance to perform internal curing without substantially impacting strength^5^. In comparison, the microcapsule cores effectively encapsulate plenty of water without introducing free water into the matrix at the beginning of cement hydration.

The stable organic shell efficiently retains the core water to ensure that each particle is in a SSD condition^23^. The size and dissolution of the microcapsules results in few defects being introduced into the microstructure of cementitious system, thereby preventing strength reduction.

Moreover, microcapsules are much smaller than LWAs, with smooth surface and standard sphere, which distributes curing water more evenly. Bentz and Snyder^24^ found that cement paste should lie within a sufficiently small distance from the internal curing water reservoir so that the internal water could efficiently penetrate. Clearly, IC agent with small inter-particle spacing promote internal curing in cement paste. Thus, it is important that IC agents are small and evenly distributed. However, small size may result in small pores that hold water tightly, reducing efficiency^22^. Thus, the structure of the IC agents is an important parameter^19^. Using a different mechanism for water release from microcapsules could solve the size issue. The main driving forces in LWAs for the migration of internal curing water is the gradient of capillary pressure between a LWA and surrounding paste matrix. migration of curing water in microcapsules depends on both the moisture gradient and the degree of shell rupture. A high IC efficiency can enhance strength and elimination of autogenous shrinkage.

Finally, microcapsules provide controlled water release over a well-defined time. In previous study, LWA typically exhibited a low efficiency for high performance concretes^25,26^. This result mainly resulted from poor porous structure and low degree of saturation of the concrete. The release of IC water from LWAs could not be controlled and either released too early or too fast. An excessively rapid release of IC water (before final setting) increase the total water-to-cement ratio. An excessively slow release of IC water maintains a reservoir of water and does not perform internal curing^4^. Thus, IC water needs to be released at an appropriate time during cement hydration. Jensen and Lura^27^ noted that the absence of method for inducing the water into the particles or ensuring the temporally appropriate release of water. In this study, the core-shell microcapsules were used to supply a possible pathway. The experimental results indicate that internal water release behaviour can be artificially controlled by tailoring the shell thickness and microcapsules size by varying the polymer dosage and stirring rate during emulsification. Thus, it can be concluded that the artificially modifiable core-shell structure is critical for the controlled release mechanism. Theoretically, water is most available for internal curing when the largest pore is the size of a particle^2^. However, among currently used IC agents, neither LWA nor SAP possess such a pore structure. Thus, in addition to optimizing the particle distribution, an IC agent should also be structurally modified to increase the IC efficiency. Table 1 is a comparison of physical characteristics and effects of microcapsules, SAPs and LAWs.

**Table 1:** Comparison of microcapsules with SABs and LWAs.

	Category	Size	Surface	Shape	Structure	Expansion in water	Autogenous shrinkage	Strength
This study	Microcapsule	44.3-115.8 μm	Smooth	Sphere	Core-shell	No	95% down	10% up
Justs^3^	SAP	250 μm (dry)	Rough	Angularity	Solid	Yes	82% down	9% down
Sensale^24^	SAP	45-150 μm (dry)	Rough	Angularity	Solid	Yes	43% down	3% down
Sensale^24^	LWA	1-2 mm	Rough	Angularity	Solid	No	57% down	16% down
Zhutovsky^22^	LWA	2.36-4.75 mm	Rough	Angularity	Solid	No	80% down	10% down

In summary, novel microcapsules have been has successfully synthesized for use as an internal curing system. These microcapsules were introduced into high-performance cementitious materials and demonstrate to controllably release internal water into a saline matrix. This new internal curing system can achieve near zero autogenous shrinkage and enhance compressive strength for high performance concrete. The microcapsules eliminate the detrimental effect of current IC agents on the compressive strength of cement paste. Moreover, this method can deliver other functional materials for high performance concrete. The microcapsule can function as intelligent carriers to change rheological behaviour, adjust the heat of cement hydration, for self-healing, for rust resistance. The novel microcapsule developed this study exhibit superior characteristics as IC agents, but are more expensive than LWA or SAB. In fact, this problem also affects the on-site application of some advanced and functional concrete materials, including for self-healing^28^, self-sealing^29^ and corrosion-resistance^30^, amongst other uses. Fabrication of composites of organic and inorganic materials may provide a feasible means of preparing microcapsule, economically. Further study by our research group is underway.

## Methods

### Fabrication of novel microcapsules

A water-in-oil-in-water (W1/O/W2) double emulsion solvent evaporation method was employed to prepare microcapsules with a water core-polymer shell structure^12^. Initially, water containing 0.1% PVA and 0.1% methylene-blue was emulsified with the CH_2_Cl_2_ solution containing 1% PMMA using a high-performance disperser 16000 HZ for 10 min. The W1/O emulsion was then poured into the outer water phase containing 0.5% PVA. The obtained W1/O/W2 emulsion was stirred continuously at 200 rpm and a temperature of 35 °C until all the CH_2_Cl_2_ evaporated. In this method, PVA was used to stabilize W1/O and O/W2 emulsion system. The volume ratios of the oil phase to the inner water phase and outer water phase to the oil phase was 5.0. Prior to characterization, the microcapsules obtained in each experiment were collected by filtration, washed with water, and dried in a fume hood. Finally, saturated surface dry (SSD) microcapsules were obtained with no surface moisture^23^. These SSD microcapsules were stored in a sealed condition at 4 °C before testing. A series of experiments was conducted to study the main factors (including polymer dosage and stirring rate) affecting the characteristics of microcapsules. In each experiment, one factor was varied while maintaining all the other factors at the designed baseline level. The baseline condition corresponded to the previous mentioned procedure. Then, different polymer dosages (1, 3, 5 and 7%) and stirring rates (200 and 300 rpm) were used to vary the shell thickness and size of the microcapsules.

### Water release behaviour test

To investigate the water release behaviour of microcapsules, methylene-blue dye was emulsified into microcapsules. In the primary emulsion(W1/O), 0.1% methylene-blue was added to water containing 0.1% PVA and emulsified with a CH_2_Cl_2_ solution containing 1% PMMA. A total of 2 g of the methylene-blue-dye microcapsules were dispersed in 50 mL of sodium hydroxide solution (pH 13) to simulate an alkaline environment for a cementitious matrix. The methylene blue concentration was measured by a UV-Vis spectrometer (Shimadzu UV-2600) to evaluate the water release behaviour. The absorbance was recorded every 15 min until a balance between the interior and exterior of microcapsules was reached, defined as the complete release of the dye/IC water.

### Characteristics of microcapsules

The morphology of the synthetic microcapsules was observed using an optical microscope (Leica MC170 HD) and a scanning electron microscope (SEM, JOEL JCM-6000 Plus). The microcapsules were fractured by freezing with liquid nitrogen and then coated with gold. Then, the shell thickness of microcapsules was evaluated by measuring at least 100 individual microcapsules in each SEM image. The microcapsule size was measured using a laser diffractometry particle size analyser (Beckman Coulter LS 13 320). The thermal stability and water content of microcapsules were characterized by a thermogravimetric analyzer (TGA, TA InstrumentsQ500). Approximately 10 mg of microcapsules were heated in a nitrogen atmosphere at a rate of 20 °C/min up to 650 °C for complete decomposition. The water content of the microcapsules was determined as the weight loss below 100 °C.

### Characteristics of mortar-microcapsule system

Table 2 shows the four mix proportions that were designed to assess the performance of the cement mortars. The water-to-binder ratio and binder-to-sand ratio were 0.4 and 1.0, respectively. The autogenous shrinkage of a mortar (D35 × L430mm) was measured by using an automated testing system based on the ASTM C1698 method^31^. The sample length was continuously recorded after final setting up to 7days with an interval of 1 min. The compressive strength of mortar-microcapsule samples (100 × 100 × 100 mm cubes) was tested at 3, 7 and 28 days by a mechanical testing machine according to EN12390-3^32^. The mortar-microcapsule samples were crushed into pieces of appropriate size for fluorescence image and SEM observation. An isothermal calorimeter (Calmetrix–I-Cal 8000) was employed to monitor the heat evolution of cement paste and the paste-microcapsule system. Each sample contained 30.0 g of Portland cement, 7.5 g of water and different weights of microcapsules (0 g, 2.7 g, 1.6 g and 2.7 g). The heat evolution in the first 72 hours was recorded continuously.

**Table 2:** Proportions of mortar mixtures.

	Cement kg/m^3^	Fly ash kg/m^3^	Sand kg/m^3^	Water kg/m^3^	SP kg/m^3^	Microcapsules kg/m^3^	IC water kg/m^3^
Control_0	954	106	1060	238	8	0	0
Control_5	954	106	957	238	8	55	0
IC_3	954	106	998	238	8	33	18
IC_5	954	106	957	238	8	55	30
